# Ferroptosis: Opportunities and Challenges in Myocardial Ischemia-Reperfusion Injury

**DOI:** 10.1155/2021/9929687

**Published:** 2021-10-23

**Authors:** Wei-kun Zhao, Yao Zhou, Tong-tong Xu, Qi Wu

**Affiliations:** ^1^Department of Health Care Ward, First Affiliated Hospital of Guilin Medical University, Guilin, Guangxi Zhuang Autonomous Region 541001, China; ^2^Department of Pathophysiology, Xuzhou Medical University, Xuzhou, Jiangsu province 221009, China; ^3^Laboratory of Clinical and Experimental Pathology, Xuzhou Medical University, Xuzhou, Jiangsu province 221009, China; ^4^Department of Physiology, Xuzhou Medical University, Xuzhou, Jiangsu province 221009, China

## Abstract

Ferroptosis is a newly discovered form of regulated cell death dependent on iron and reactive oxygen species (ROS). It directly or indirectly affects the activity of glutathione peroxidases (GPXs) under the induction of small molecules, causing membrane lipid peroxidation due to redox imbalances and excessive ROS accumulation, damaging the integrity of cell membranes. Ferroptosis is mainly characterized by mitochondrial shrinkage, increased density of bilayer membranes, and the accumulation of lipid peroxidation. Myocardial ischemia-reperfusion injury (MIRI) is an unavoidable risk event for acute myocardial infarction. Ferroptosis is closely associated with MIRI, and this relationship is discussed in detail here. This review systematically summarizes the process of ferroptosis and the latest research progress on the role of ferroptosis in MIRI to provide new ideas for the prevention and treatment of MIRI.

## 1. Introduction

Cell death is the natural endpoint of typical cells, occurring in growth and development, division and differentiation, and homeostatic metabolism, ultimately resulting in the irreversible end of the cellular function. The mode of cell death in the process of myocardial ischemia-reperfusion injury (MIRI) has been garnering substantial attention. Although cell death primarily constitutes apoptosis and necrosis, ferroptosis, a new form of programmed cell death that is iron-dependent and distinct from apoptosis and necrosis, has been discovered in recent years [1–3]. In recent years, ferroptosis has received extensive attention because it participates in the pathophysiological processes of tumor formation, kidney-related diseases, neurodegenerative diseases, stroke, and other diseases [4]. The occurrence and development of ferroptosis are closely related to the pathological process of myocardial cells, with ferroptosis participating in the pathogenic mechanisms of MIRI [5]. Ferritin was found to accumulate at the myocardial scar area of the left anterior descending coronary artery of mice in the ischemia-reperfusion injury(IRI) model after 30 min of ligation [6]. In addition, an in vitro study with perfused hearts showed that ferroptosis is crucial for the pathogenic mechanism of IRI; deferoxamine, a chelating agent, can prevent isolated hearts from IRI [7]. Erastin, an agonist of ferroptosis, can inhibit cystine ingestion and the downstream synthesis of glutathione, leading to an imbalance in cellular redox and, thus, cell death. In contrast, ferroptosis inhibitors can effectively alleviate organ injury induced by ischemic reperfusion [8]. Ferroptosis is a type of cell death in the pathogenic process of MIRI, and its obstruction can lead to substantial protection of myocardial cells. In this paper, we focus on the recent research progress between ferroptosis and MIRI and discuss the important role of ferroptosis in the regulation of MIRI.

## 2. Ferroptosis

**Figure 1:**
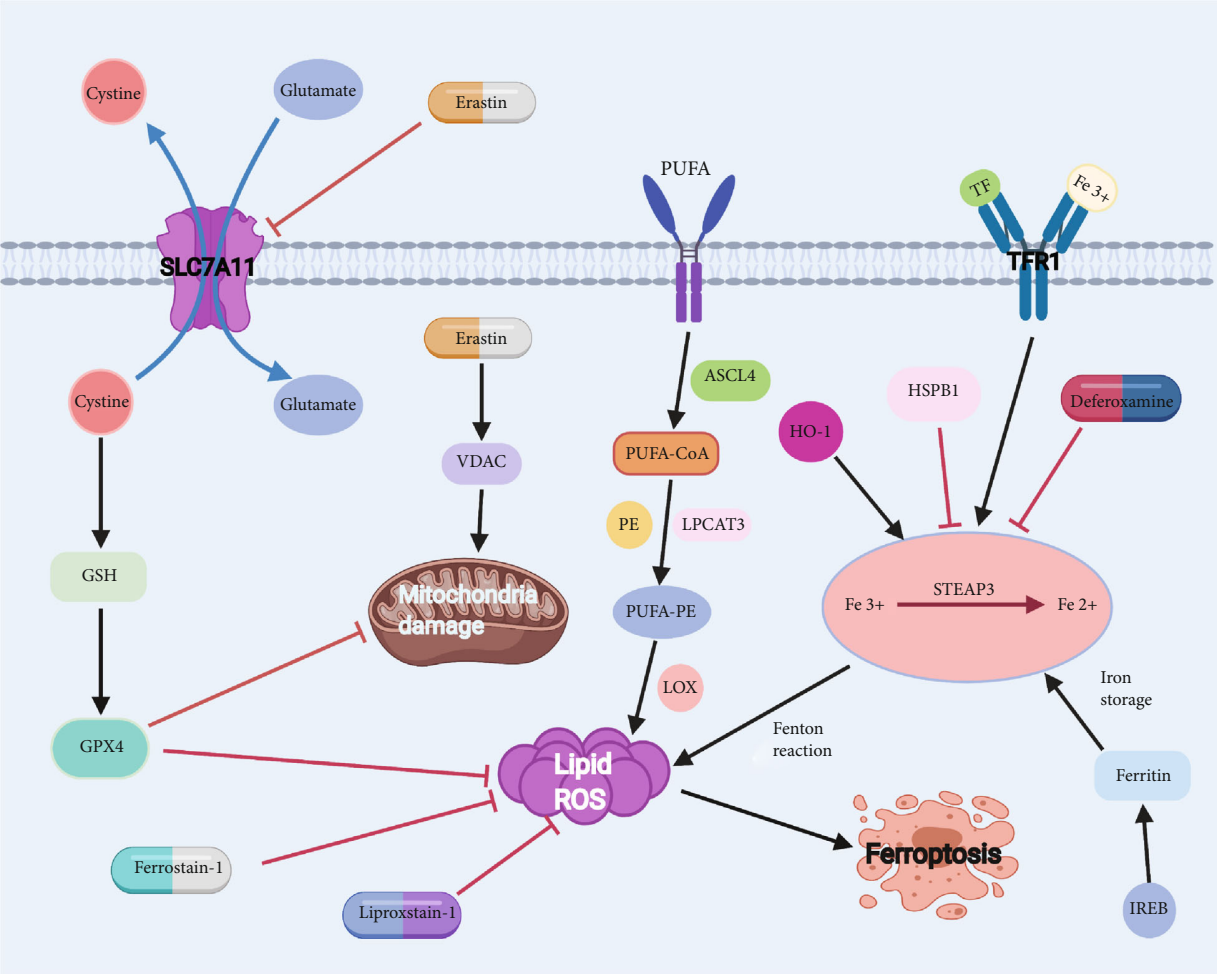
Cartoon depicting the possible mechanism and regulation of ferroptosis. Ferroptosis is mainly regulated by Fe homeostasis, lipid oxidation, System Xc^−^, and VDAC. The most important in the ferroptosis signaling pathway is the production of iron ions and ROS. GPX-4 is a key regulator of ferroptosis. Inhibition of GPX-4 causes a large amount of lipid peroxides to aggregate, becoming a sign of ferroptosis.

Early studies demonstrated that erastin and RAS-selective lethal compound 3 (RSL3) could cause cell death in a manner that is different from apoptosis, which can be inhibited by iron chelators and antioxidants [9–11]. In 2012, after continuous exploration and discussion of nonapoptosis cell death processes, Dixion and coworkers proposed an iron-dependent nonapoptosis cell death process, now known as ferroptosis [8]. This process involves the iron-dependent accumulation of reactive oxygen species (ROS) that exceeds the cell's ability to maintain redox homeostasis, leading to lipid peroxidation and eventually causing cell death. Glutathione peroxidase-4 (GPX-4) is a key regulatory protein in ferroptosis [1]. The main morphological manifestations of ferroptosis are mitochondrial abnormalities, including reduced mitochondrial volume, mitochondrial cristae dissolution, and increased mitochondrial membrane density and rupture. However, whether mitochondrial damage can be reversed during the process of ferroptosis remains controversial [12]. The biochemical characteristics of ferroptosis mainly include the accumulation of iron and ROS, glutathione (GSH) depletion, the release of arachidonic acid, and inhibition of the cystine/glutamate antiporter system (System Xc-) pathway [13]. Due to continuous research, a preliminary understanding of the process of ferroptosis began to take shape, and it has now been shown that ferroptosis is mainly regulated by multiple intracellular signaling pathways, such as iron homeostasis regulation, lipid peroxidation, System Xc-, and the voltage-dependent anion channel (VDAC) pathway [14–17] (Figure 1).

## 3. Ferroptosis Process

### 3.1. Regulatory Pathways of Iron Homeostasis

The maintenance and regulation of iron homeostasis are extremely complex processes. Iron is one of the most important essential trace elements in the human body and is involved in a series of important biological processes. Iron ions exist as Fe^2+^ and Fe^3+^ in the body and can be considered a “double-edged sword.” The element is also the primary raw material for the synthesis of hemoglobin and myoglobin and is crucial for important processes, such as electron transport, cellular respiration, DNA synthesis, cell proliferation and differentiation, and gene regulation. However, the excessive accumulation of iron ions can increase ROS production through the Fenton reaction, which affects iron stability and promotes iron deposition in vital organs, thereby leading to severe organ damage.

In the process of iron metabolism, divalent metal transporter-1 (DMT-1) is a key protein in the intracellular transport of iron. Fe^3+^ bound to transferrin enters the cell via the membrane protein, transferrin receptor-1 (TFR-1), to form endosomes. Free Fe^3+^ is reduced to Fe^2+^ in the endosomes by the metal reductase, six-transmembrane epithelial antigen of the prostate-3 (STEAP-3). Fe^2+^ is then transported from the endosomes to the labile iron pool in the cytoplasm under the mediation of DMT-1. This is the process of iron recycling [18]. Ferritin is involved in this process as a complex of iron storage proteins consisting of ferritin light chain (FTL) and ferritin heavy chain-1 (FTH-1) and participates in the regulation of iron ions as a multimer. FTH-1 has iron oxidase activity, which catalyzes the conversion of Fe^2+^ to Fe^3+^ and stores it in ferritin molecules, thereby reducing free iron levels, while FTL is directly involved in iron storage. In addition, heme oxygenase-1 (HO-1) was found to lead to ferroptosis by increasing intracellular iron and mediating lipid peroxidation reactions [19]. When intracellular iron homeostasis is disrupted, excess iron converts hydrogen peroxide and lipid peroxides to ROS via the Fenton reaction, which in turn causes ferroptosis [4, 20].

Various iron regulatory proteins are also involved in the process of iron metabolism. Iron-responsive element-binding protein-2 (IREB-2) also has an important function, which involves the enhanced expression of FTL and FTH-1, leading to decreased levels of intracellular iron and the inhibition of erastin-induced ferroptosis. This implies that the IREB-2 expression can indirectly interfere with iron adsorption and inhibit ferroptosis [1, 21]. In addition to IREB-2, recent studies have revealed that heat shock protein B-1 (HSPB-1) may also play a key role as an iron regulatory protein in iron metabolism. HSPB-1 can inhibit TFR-1, which results in lower iron ion concentration, thereby further suppressing the occurrence of ferroptosis [22]. Therefore, the regulation of iron homeostasis plays an important role in the process of ferroptosis.

### 3.2. Lipid ROS Production

The formation of lipid ROS is a key component in the onset and development of ferroptosis. ROS production depends on the action of polyunsaturated fatty acid-phosphatidyl ethanolamine (PUFA-PE). PUFA can be acylated under the catalysis of acyl-CoA synthetase long-chain family member-4 (ACSL-4) to produce PUFA acyl-CoA (PUFA-CoA), which then reacts with PE under the action of lysophosphatidylcholine acyltransferase-3 (LPCAT-3) to produce PUFA-PE [16, 23, 24]. Under the enzymatic catalysis of lipoxygenase (LOX), PUFA-PE is essential for the formation of ROS [14, 17].

In this pathway, the action of PUFA-PE depends on two key regulatory points, ACSL-4 and LOX. Therefore, decreasing ACSL-4 and LOX can effectively inhibit the action of PUFA-PE and suppress the onset of ferroptosis [25]. The knockdown of the ACSL-4 gene in breast cancer cells can lead to a significant reduction in PUFA-PE production and suppress ferroptosis [26]. Furthermore, thiazolidinediones specifically inhibit ACSL-4, thereby suppressing ferroptosis [23, 27]. Zileuton (a 5-LOX inhibitor) can also inhibit erastin and ferroptosis in HT22 neuronal cells [28]. In addition, ROS formation is jointly achieved by a combination of iron-mediated Fenton reaction, the System Xc^−^ pathway, and the VDAC pathway, which ultimately leads to ferroptosis.

### 3.3. System Xc^−^ Pathway

System Xc^−^ is an amino acid transporter expressed in the plasma membrane of mammalian cells. It is a heterodimer composed of SLC7A11 and SLC3A2, acting primarily through SLC7A11 (the primary active site of erastin). System Xc- exchanges extracellular cystine (Cys) for intracellular glutamate (Glu) at a 1 : 1 ratio, thus providing the raw material for intracellular GSH synthesis. Cellular uptake of cysteine is an important step in GSH production [29, 30]. GPX-4 is a GSH-dependent enzyme that converts GSH into oxidized glutathione (GSSG), which in turn can scavenge excess peroxides and hydroxyl radicals produced during cellular respiration and metabolism. Thus, GPX-4 plays an indispensable role in preventing lipid peroxidation [31]. When GPX-4 function is restricted, this is often accompanied by a decrease in GSSG and a significant increase in ROS [32]. Furthermore, the inhibition of GPX-4 activity will promote ROS formation and lipid peroxidation, thereby leading to ferroptosis. SLC7A11 gene silencing with siRNA interference substantially increased the sensitivity of HT-1080 cells to erastin-induced ferroptosis, while the overexpression of SLC7A11 in HT-1080 cells significantly enhanced cellular resistance to ferroptosis [33]. Another study found that the tumor suppressor P53 downregulated SLC7A11, inhibited System Xc- uptake of Cys, decreased GPX, increased ROS, and ultimately induced ferroptosis [34]. In addition, nicotinamide adenine dinucleotide phosphate (NADPH) was shown to maintain GSH in a reduced state, which further regulates ferroptosis [35]. Therefore, the inhibition of the System Xc- pathway will reduce intracellular GSH levels, resulting in decreased GPX-4 activity, which will ultimately lead to ferroptosis [30].

### 3.4. VDAC Pathway

VDAC is a channel protein for transporting ions and metabolites located on the outer mitochondrial membrane and consists of VDAC-1, VDAC-2, and VDAC-3. It controls the exchange of metabolites in the mitochondria and with other organelles [36]. In addition to regulating mitochondrial metabolism and energy production functions, VDAC can also potentially regulate cell survival and death signals by interacting with different ligands and proteins. Erastin, a typical inducer of ferroptosis, can activate VDAC in the presence of tubulin and cause mitochondrial hyperpolarization, thus leading to ROS production, mitochondrial dysfunction, and cell death [37]. When the VDAC-2 or VDAC-3 expression was inhibited by siRNA interference, cells were tolerant to erastin-induced ferroptosis. However, the overexpression of VDAC-2 or VDAC-3 did not increase cellular sensitivity to erastin. Therefore, VDAC may participate in cellular ferroptosis. In addition, mitochondria are key targets of the MIRI mechanism. The opening of the mitochondrial permeability transition pore (mPTP) can lead to elevated mitochondrial ROS production, membrane potential loss, and ATP depletion, thereby inducing cell death through mechanisms of programmed or nonprogrammed death. Meanwhile, erastin action on VDAC alters the permeability of the outer mitochondrial membrane, thus causing mitochondrial dysfunction, increased ROS production, and ultimately cellular ferroptosis [38–40]. Therefore, the VDAC pathway and mPTP may be involved in the mechanism of MIRI.

### 3.5. Other Related Signaling Pathways

At present, further indepth investigations have led to the gradual recognition of the roles of NADPH oxidase-4 (NOX-4), HSPB-1, and other related proteins and signaling pathways in the regulation of ferroptosis [22, 41]. The mechanisms of ferroptosis are shown in Figure 1.

## 4. The Role of Ferroptosis in MIRI

### 4.1. Ferroptosis and MIRI

Myocardial ischemia caused by coronary artery obstruction is clinically manifested as persistent severe retrosternal pain and can lead to myocardial infarction, shock, arrhythmia, or heart failure. The most common treatment strategy is the early restoration of blood flow to the ischemic area using techniques, such as coronary angioplasty, percutaneous coronary intervention, and coronary artery bypass grafting (CABG), which can restore myocardial oxygen and nutrient supply, salvage the ischemic myocardium, and save the patient's life.

MIRI is a phenomenon wherein cardiac function does not improve but worsens immediately after perfusion is restored to the ischemic myocardium. The pathogenic mechanism of MIRI is not fully understood. At present, it is known to primarily be involved in processes such as oxidative stress, calcium overload, and in inflammatory reactions [42]. Oxidative stress can lead to cell membrane rupture, swelling, or death through intracellular homeostasis, mitosis, cellular differentiation, and intracellular signaling [43]. As research progresses, ferroptosis has been identified as a form of cell death in MIRI pathogenesis that is closely related to oxidative stress. At present, peroxidized phosphatidylethanolamine (PEox) has been identified as a predictive biomarker of ferroptosis, and Sparvero's group was the first to apply gas cluster ion beam secondary ion mass spectrometry (GCIB-SIMS) as a technique to increase PEox in cardiomyocytes, which provided direct evidence for the occurrence of ferroptosis in cardiomyocytes [44]. Iron chelating agents can bind to iron ions in the plasma or tissues and promote their elimination via urea or bile, thereby reducing the iron content in the body. Deferoxamine is a frequently used iron-chelating agent. Furthermore, in a study of patients with coronary artery disease (CAD), when performing CABG, the intravenous infusion of deferoxamine 8 h after anesthesia was effective in ameliorating oxygen radical production and protecting the myocardium from reperfusion injury, with more pronounced benefits in patients with reduced left ventricular ejection fraction (LVEF) [45]. A previous study also showed that using nuclear magnetic resonance spectroscopy in perfusion experiments on isolated rabbit hearts and adding a certain concentration of deferoxamine at the early stage of perfusion could effectively attenuate reperfusion-induced free radical generation, thus achieving cardioprotective effects [46]. Most current studies investigating the role of ferroptosis in MIRI have mainly focused on endoplasmic reticulum stress (ERS) and ROS production, GPX-4, and the autophagy-dependent ferroptotic pathway.

#### 4.1.1. ERS

Ferroptosis occurs with the production of ERS, and ERS-induced unfolded protein response plays an important role in the ferroptotic process. When changes in the calcium level and redox status of the endoplasmic reticulum (ER) lumen induce a decline in chaperone protein function, cells can activate the unfolded protein response and cause ERS. ERS disrupts Ca^2+^ homeostasis in the ER, leading to mitochondrial calcium overload and elevated ROS production, while the accumulation of ROS will activate downstream caspase family proteins through cascade amplification, thereby initiating the process of cellular damage. In addition, the ERS process is induced by upstream signaling proteins, including inositol-requiring enzyme-1, activating transcription factor-6 (ATF-6), and PKR-like ER kinase (PERK) [47]. The ERS response elicited by ferroptosis inducers plays a tandem role between ferroptosis and other types of cell death [48], mainly in the form of ERS-mediated activation of the PERK-eukaryotic initiation factor 2*α* (eIF2*α*)-ATF 4-CHOP pathway. The dissociation of PERK from binding immunoglobulin protein BiP will trigger the phosphorylation and subsequent activation of PERK. Furthermore, eIF2*α* is activated, leading to ATF 4 mRNA translation and the induction of downstream CHOP molecules. The CHOP-mediated apoptosis in ERS plays an important role in the MIRI process in rats [49].

Ferroptosis induces ERS-triggered apoptosis. Studies have found that ferroptosis can induce ERS activation by inhibiting the System Xc--mediated exchange of extracellular cystine for intracellular glutamate [50, 51]. The activation of the PERK- eIF2*α*-ATF 4 pathway accompanying the ESR response regulates the target gene of the unfolded protein response, CHOP, while the binding of CHOP to the corresponding promoter inducesthe expressions of PUMA, endoplasmic reticulum redox protein-1*α*, and B-cell lymphoma-2 (Bcl-2) [52, 53]. Furthermore, ferroptosis agonists can induce the PUMA expression but not the Bcl-2 expression, suggesting that the ferroptosis-induced PUMA gene expression was unable to induce apoptosis [54]. In addition, tumor necrosis factor- (TNF-) related apoptosis-inducing ligand (TRAIL) binds to the corresponding death receptors to form a death-inducing signaling complex, which in turn induces apoptosis. With the help of the death-inducing signaling complex, caspase-8 is activated, which can lead to the further activation of caspase-3, caspase-6, and caspase-7, eventually resulting in apoptosis. Ferroptosis agonists also modulate the cellular activity induced by TRAIL [54]. The interaction between ferroptosis and the apoptotic molecule TRAIL can be mediated by the ERS-induced expression of PUMA molecules. This suggests that ferroptosis-induced ERS can act as a bridge between ferroptosis and apoptosis. MIRI has also been found to be closely associated with ERS [55].

Apoptosis results from ferroptosis-induced ERS and its correlation with MIRI. In a rat MIRI model, it was found that erastin probably increases ERS, which further increases ferroptosis. Inhibition of ferroptosis can reduce myocardial cell injury, and the inhibition of ERS can alleviate ferroptosis and reduce MIRI. These findings suggest that ferroptosis is involved in ERS-associated MIRI. Furthermore, based on a MIRI model established by ligating the left anterior descending branch of the coronary artery in diabetic rats, the tail vein injection of the ferroptosis inhibitor, ferrostatin-1, could attenuate ERS-induced ferroptosis in cardiomyocytes, while the ERS inhibitor, salubrinal, could also attenuate ferroptosis in cardiomyocytes. This suggests that the activation of ERS may exacerbate the process of ferroptosis in cardiomyocytes, while the occurrence of ferroptosis can further exacerbate ERS in cardiomyocytes, which can form a vicious circle [56]. Therefore, ferroptosis-induced ERS and the activation of ERS play crucial roles in apoptosis and are important apoptotic mechanisms in MIRI.

#### 4.1.2. GPX-4

GPX-4 participates in the regulation of ferroptosis. GPX-4 is an endogenous antioxidant for selenium-dependent enzymes that serves as a core regulator in the ferroptotic signaling pathway. Under physiological conditions, GPX-4 can confer cellular protection by scavenging lipid peroxides, thus preventing iron-mediated lipid peroxidation and elevated lipid ROS, whereas GPX inactivation induces ROS lipid peroxidation and ferroptosis [57]. System Xc- mediates cystine uptake and glutamate release to promote GSH synthesis, while GSH acts as a synergistic molecule of GPX-4 to assist in scavenging lipid peroxides for cellular protection. Erastin inhibits System Xc- and indirectly inactivates GPX-4, thus leading to the accumulation of lipid peroxides to promote the onset of ferroptosis [58]. The indirect or direct inactivation of GPX-4 is the classic induction mechanism of ferroptosis. ML162 and RSL3 induce ferroptosis by depleting GPX-4 [59]. In addition, GSH participates as a coenzyme in the breakdown of hydrogen peroxide by GPX-4; therefore, by inhibiting the intracellular activity, GSH and GPX-4 increase intracellular ROS levels and ultimately mediate ferroptosis.

GPX-4 mediates ferroptosis to regulate MIRI. A previous study demonstrated that the levels of iron and malondialdehyde (MDA) in reperfused rat hearts gradually increased with increasing reperfusion time, accompanied by a decrease in GPX-4 levels [60]. Notably, there is evidence showing that the specific overexpression of GPX-4 in mitochondria attenuates cardiac dysfunction in MIRI [61]. Furthermore, GPX-4 is involved in the pathogenesis of MIRI. GPX-4 is an important antioxidant enzyme upstream of the mitochondria that regulate ferroptosis and oxidative stress by catalyzing the conversion of reduced glutathione (GSH) to oxidized glutathione (GSSG). Moreover, the knockdown of GPX-4 in a glutathione-independent manner leads to the destruction of mitochondrial morphology and increased mitochondrial ROS production [62]. In turn, the large production of ROS and ferroptosis are important mechanisms leading to MIRI [63, 64]. Another study found that liproxstatin-1 inhibited ferroptosis by increasing GPX-4 levels, which decreased ROS levels and thus alleviated MIRI [65]. Therefore, in MIRI, increasing GPX-4 expression can inhibit ferroptosis and attenuate the negative effects of MIRI.

#### 4.1.3. ROS

Pathogenic mechanism of ROS participation in MIRI. Oxidative stress, attributed to the enhanced production of ROS during MIRI, is the main cause of MIRI. Excessive ROS accumulation causes membrane lipid peroxidation and disrupts the barrier function of the cell membrane. Excessive oxidation of lipids, DNA, and proteins causes increased cardiomyocyte damage and ultimately cell death. The antioxidant system regulates redox homeostasis by controlling intracellular ROS levels and the interactions between normal cellular metabolism and pathophysiology. Increased expression of antioxidant enzymes protects tissues from oxidative stress and produces cardioprotection after myocardial reperfusion [66]. During reperfusion, myocardial tissue is reoxygenated as blood flow is restored with a sudden increase in ROS production during the first few minutes, which is one of the underlying pathogenic mechanisms causing MIRI.

Increasing ROS levels lead to ferroptosis. Ferroptosis occurs due to increased intracellular iron concentration and the depletion of the antioxidant GSH, which leads to increased levels of ROS and in turn causes lipid peroxidation and eventually cell death. It was found that lipid peroxidation may occur in the lysosomal membrane due to ROS accumulation and iron overload, while the permeabilization of the lysosomal membrane can lead to oxygen radical production, cell membrane degeneration, and increased GSH depletion [67]. Moreover, lysosomes can regulate iron homeostasis and cause a dramatic increase in ROS expression. In addition, under the participation of iron ions, ROS are produced in a nonenzymatic pathway. For example, free iron ions present in the unstable iron pool form Fe^3+^ and hydroxyl radicals in the presence of Fe^2+^ and H_2_O_2_ through the Fenton reaction. Alternatively, Fe catalyzes the production of -OH through the Haber-Weiss reaction. The inhibition of GPX-4 causes an increase in ROS, whereas the overexpression of GPX-4 reduces ROS and thus cellular ferroptosis [62, 68]. The increase in ROS leads to lipid peroxidation and ferroptosis, which can be inhibited by the iron chelator deferoxamine [69]. On the other hand, higher levels of iron transporters will increase iron-mediated ROS, which subsequently leads to ferroptosis. Liproxstatin-1 has been shown to significantly inhibit ferroptosis and attenuate MIRI by reducing the accumulation of ROS from lipid peroxidation, protecting the structural integrity of mitochondria, increasing the levels of the GPX-4 protein, and reducing ROS levels [70].

ROS play a role in MIRI-mediated ferroptosis. Ferroptosis is highly correlated with cardiomyocyte death. During MIRI, iron accumulates in cardiomyocytes around the myocardial scar, and excess iron leads to cardiomyocyte death, while the inhibition of ROS production attenuates cardiomyocyte death [6]. Based on in vivo and in vitro models of MIRI, ROS levels were significantly elevated in MIRI myocardial tissues, while sirtuin-1 (SIRT-1) and SLC7A11 expression were downregulated, and p53 was highly expressed. Following the overexpression of SIRT-1, the cardiomyocytes showed a significant improvement in the extent of ferroptosis, reduction in ROS levels, upregulation of the SLC7A11 protein expression, and downregulation of the p53 protein expression. This suggests that ROS plays an important role in MIRI ferroptosis, and that the regulation of ROS may be related to the SIRT-1/p53/SLC7A11 signaling pathway [71].

#### 4.1.4. Cellular Autophagy

Autophagy is an important mechanism of MIRI. Autophagy is a precisely regulated, dynamically developing process that cleans up damaged organelles and proteins via lysosomes, and is a monitoring mechanism that is relatively conserved. It recycles the basic nutrients produced and plays an important role in maintaining the normal structure and function of the heart. This involves not only cell survival but also cell death [72, 73]. In MIRI, the molecular mechanism of autophagy consists mainly of mammalian target of rapamycin (mTOR) and beclin 1, which play indispensable roles at different phases of MIRI. mTOR exerts its effects in the myocardial ischemic phase by mediating AMPK/mTOR and PI3K/Akt/mTOR signaling pathway [74] and further in the reperfusion phase via the upregulation of the beclin 1 pathway [72, 75, 76]. The possible mechanisms of beclin 1 activation in MIRI mainly include the following: (1) the association of beclin 1 with the Bcl-2 protein. (2) ROS can act as an inducer to mediate beclin 1 autophagy during reperfusion injury. (3) ROS can decrease the activity of autophagy-associated proteins through oxidation, thus causing LC 3 lipidation and autophagy. Therefore, autophagy is involved in MIRI [77].

Ferroptosis is closely related to autophagy. To a certain degree, ferroptosis is dependent on autophagy and involves the embryonic lethal abnormal vision-like protein-1 (ELAVL-1) and forkhead box C-1 (FOXC-1) [78]. ELAVL-1 is a protein-coding gene that regulates the gene expression by stabilizing RNA (TNF-*α* or VEGF-A) and is implicated in the processes of apoptosis and oxidative stress [79]. ELAVL-1 inhibits the inflammatory response in AMI and plays a role in MIRI, where the significant increase in ELAV-1 is accompanied by the excessive production of ROS and inflammatory cytokines [63, 80]. FOXC-1 plays a significant role as a transcription factor in cell growth and survival, as well as in cardiac disease [81]. FOXC-1 transcription activates ELAVL-1, and the ELAVL-1-mediated enhancement of the autophagic ferroptosis pathway has a significant impact on MIRI. During MIRI, decreased GSH and GPX-4 levels can lead to elevated ELAVL-1, which further inhibits enzyme function and cellular antioxidant capacity. ELAVL-1 also inhibits ferroptosis and MIRI, restores GPX-4 expression level, restores cardiomyocyte viability, and attenuates cardiomyocyte injury. Low levels of ELAVL-1 can inhibit MIRI-induced autophagy, suppress ferroptosis, and attenuate myocardial infarct size and MIRI. In addition, a decrease in the FOXC-1 expression was followed by a decrease in ELAVL-1 level, suggesting that FOXC regulates the ELAVL-1 expression during MIRI. Thus, autophagy-dependent ferroptosis counteracts the effects of reduced ELAVL-1 and contributes to the onset of MIRI and the overproduction of lipid signaling. Therefore, the relationship between FOXC-1 and ELAVL-1, as well as its association with ferroptosis, could serve as useful targets against MIRI.

Autophagy regulates ferroptosis to participate in the pathogenic mechanism of MIRI. Previous studies showed that ferroptosis is different from cellular autophagy and other modes of cell death, whether in terms of cell morphology, biochemical characteristics, or the regulatory factors involved. However, recent studies have revealed an interconnection between autophagy and ferroptosis in cardiomyocytes during the course of MIRI, wherein the activation of ferroptosis depends on the induction of autophagy, and the regulatory proteins of autophagy may also be involved in the regulation of ferroptosis. In another study, iron ion levels and ROS were significantly enhanced in the cardiomyocytes of MIRI rats, whereas GPX-4 and GSH protein expressions were significantly reduced, thus suggesting that ferroptosis may be involved in the pathogenesis of MIRI. Further studies revealed that myocardial ferroptosis may be regulated by autophagy-related signaling pathways during the progression of MIRI, and the ELAVL-1 protein is able to bind specifically with the autophagy-related protein beclin 1 to promote the decrease in the P62 protein expression and increase in LC 3 levels. This will induce an increase in the autophagy levels of cardiomyocytes and thus activate the ferroptotic pathway [80]. At present, indepth studies have been conducted on the role of autophagy in ferroptosis in fields, such as cancer. However, in MIRI, most studies have only superficially concluded that ferroptosis in cardiomyocytes may be regulated by autophagy, while its underlying mechanisms of action remain poorly understood. Therefore, we hope that more indepth studies will be conducted in this area in the future, so as to achieve new breakthroughs in the treatment of MIRI.

### 4.2. Treatment

There is currently no effective treatment for MIRI. With the advancement of research, researchers began identifying the inhibition of ferroptosis cardiomyocytes as a potentially important target for the treatment of MIRI [12]. Pretreatment of MIRI mice with ferrostatin-1 (Fer-1, an inhibitor of ferroptosis) or dexrazoxane (an iron chelating agent) significantly increased the expression level of Ptgs2 mRNA, which further led to a reduction in the myocardial enzyme spectrum and the scar area of myocardial infarction [64]. In contrast, liproxstatin-1 (Lip-1) treatment maintained the structure and function of mitochondria after MIRI by reducing VDAC-1 levels and restoring GPX4 protein levels [70]. Overexpression of USP22 and inhibition of glutaminase can alleviate MIRI by inhibiting ferroptosis [7, 71]. In a MIRI rat model and oxygen–glucose deprivation/reoxygenation (OGD/R) H9c2 cells, it was found that ACSL4-mediated ferroptosis was a promising target for MIRI treatment, and baicalin can prevent MIRI by inhibiting ACSL-4-mediated ferroptosis [82].

A recent study found that dexrazoxane or ponatinib inhibited ferroptosis during MIRI, and a combined treatment with both drugs markedly reduced the scar area of myocardial infarction. However, for myocardial infarction patients with an elevated ST section, percutaneous coronary intervention before myocardial reperfusion could not significantly reduce the scar area of myocardial infarction [83]. Based on these findings, a combined treatment targeting different types of cell death is proposed as an effective treatment strategy for MIRI.

Recently, it was proven that phosphatidylcholine oxide content increased dramatically during MIRI, leading to reduced GPX-4 activity and ferroptosis. However, it is noteworthy that Fer-1 can inhibit OxPC-triggered ferroptosis [84]. Another study showed that the expression level of ELAVL-1 was upregulated. ELAVL-1 can also be activated by the autophagy-regulated ferroptosis process, which is related to FOXC-1 transcription, and ELAVL-1 knockout can reduce ferroptosis and alleviate MIRI [80]. Cyanidin-3-glucoside (C3G) treatment can effectively alleviate the expression of proteins related to apoptosis, reduce Fe^2+^ content, and improve MIRI. Therefore, C3G is a potential medicine to prevent myocardial cells from being affected by MIRI [85].

Ferroptosis has been shown to be related to diabetic MIRI. Fer-1 alleviates ERS, reduces cellular injury in H9c2 cells, and mitigates myocardial cell injury during diabetic MIRI. In addition, diabetes patients can induce MIRI by activating NOX-2-related oxidative stress and apoptosis, and inhibition of nicotinamide adenine dinucleotide phosphate oxidase-2 (NOX-2) can alleviate MIRI in diabetic rats [56, 86]. Fer-1 can also mitigate myocardial cell injury in H9c2 cells under hyperglycemic conditions and reduce H9c2 cell injury during anoxia/aeration. These results have provided beneficial treatment for patients with diabetic MIRI.

Additionally, further understanding of the association between ferroptosis and MIRI after heart transplantation has been obtained. Fer-1 reduces myocardial cell death and blocks the recruitment of neutrophil granulocytes to damaged myocardial cells by damage-associated molecular patterns (DAMPs) after heart transplantation [87]. Therefore, targeted ferroptosis can potentially provide preventative treatment of MIRI in patients undergoing heart transplantation after coronary artery reperfusion. Other drugs, such as piperlongumine, isothiocyanates, and artemisinin, may exert cardiomyocyte protective effects by inhibiting ferroptosis. However, articles related to the effects of Chinese medicines and other drugs on MIRI are still scarce, which warrants further investigation (see Table 1 for details).

## 5. Conclusion

Ferroptosis is an iron-dependent, nonapoptotic mode of cell death characterized by ROS accumulation. However, current research on ferroptosis is still in its infancy. Ferroptosis is closely associated with MIRI and regulates MIRI through ERS, ROS production, GPX-4, and the autophagy-dependent ferroptotic pathways. Ferroptosis can serve as an important target in MIRI, which may help in the process of reducing the occurrence of MIRI. Therefore, indepth studies on ferroptotic mechanisms and possible interventions have now become a focus of current research. In addition, the precise stage of MIRI at which ferroptosis mainly occurs have not been located, and some studies have shown that the incidence of ferroptosis is different at different stages of MIRI [99]. Therefore, more indepth investigations will provide new ideas for the prevention and treatment of MIRI.

## Figures and Tables

**Table 1:** Small molecules, drugs, and ferroptosis.

Small molecules or drugs	Intervention target	Molecular weight	Molecular formula	Function	Experimental cells/animals	References
Erastin	VDAC 2/3 or system Xc^−^	547.04	C30H31ClN4O4	Prevents cystine import, causes GSH exhaustion,cause ferroptosis	BJeLR HT1080 143B p° and p^+^ cell U2OS DU-145	[1, 9, 10, 51, 88]
RSL3	GPX-4	440.9	C23H21ClN2O5	Covalent inhibitor of GPX-4 that causes accumulation of lipid hydroperoxides and ferroptosis	KBM7 MIA PaCa-2 A549, Calu-1, HCT116, HT1080 BJeLR	[10, 89]
Buthionine sulfoximine	GSH exhaustion	222.305	C8H18N2O3S	Cause ferroptosis	BJeLR HCT116/A549	[51]
Acetaminophen	GSH exhaustion	151.163	C8H9NO2	Cause ferroptosis	HepG2/primary mouse	[90]
Sulfasalazine	System Xc^−^	398.394	C18H14N4O5S	Low potency inhibitor that prevents cystine import, causes GSH depletion and ferroptosis	BJeLR/HT1080 HT1080/Calu-1	[1, 51]
Sorafenib	System Xc^−^	464.825	C21H16ClF3N4O3	Cause ferroptosis	HT1080/Calu-1DU-145 nude mice	[51, 91–93]
Artesunate	—	384.421	C19H28O8	Cause ferroptosis	PDAC cell lines	[94]
Piperazine erastin	VDACs or system Xc^−^	645.19	C35H41ClN6O4	Cause ferroptosis	BJeLR nude mice	[88]
Trolox	Lipophilic antioxidants	250.29	C14H18O4	Blocks propagation of lipid peroxidation, may inhibit lipoxygenases, inhibiting ferroptosis	HT1080/PUFA oxidation-induced death model on S. cerevisiae	[1, 95]
Ebselen	Oxidative pathway	274.17666	C13H9NOSe	Inhibiting ferroptosis	HT1080, Calu-1	[1]
SSRS 11-92	ROS from lipid peroxidation	—	—	Inhibiting ferroptosis	HD model	[95]
*α*-Tocopherol (vitamin E)	Oxidative pathway	430.71	C29H50O2	Blocks propagation of lipid peroxidation, may inhibit lipoxygenases, inhibitting ferroptosis	BReLR GPX4-deficient T-cell mice	[10, 96]
Deferoxamine	Fenton reaction	560.68	C25H48N6O8	Depletes iron and prevents iron-dependent lipid peroxidation, inhibiting ferroptosis	Wild-type and Bax/Bak	[1]
Deferoxamine mesylate (DFO)	Intracellular iron	656.8	C25H48N6O8•CH4O3S	Inhibiting ferroptosis	BJeLR	[10]
SRS 16-86	ROS from lipid peroxidation	432.2525	C16H24N2O2	Inhibiting ferroptosis	HT1080/NIH 3T3 IRI mice model	[97]
Ferrostatin-1 (Fer-1)	ROS from lipid peroxidation	262.35	C15H22N2O2	Blocks lipid peroxidation, inhibiting ferroptosis	HT1080	[1, 95]
Liproxstatin-1 (Lip-1)	ROS from lipid peroxidation	340.85	C19H21ClN4	Blocks lipid peroxidation, inhibiting ferroptosis	HRPTEpiCs GPX4^−/−^ cells GPX4^−/−^ mice	[98]

## Data Availability

The data used to support the finding of this study are available from the corresponding author upon request.

## Abbreviations

ACSL-4: Acyl-CoA synthetase long-chain family member-4
AMI: Acute myocardial infarction
ATF-6: Activating transcription factor 6
Bcl-2: B-cell lymphoma-2
CABG: Coronary artery bypass grafting
CAD: Coronary artery disease
Cys: Cysteine
C3G: Cyanidin-3-glucoside
DAMPs: Damage-associated molecular patterns
DMT-1: Divalent metal transporter 1
eIF2*α*: Eukaryotic initiation factor
ELAVL-1: Embryonic lethal-abnormal vision like protein 1
ER: Endoplasmic reticulum
ERS: Endoplasmic reticulum stress
Fer-1: Ferrostatin-1
FOXC-1: Forkhead box C-1
FTH-1: Ferritin heavy chain-1
FTL: Ferritin light chain
GCIB-SIMS: Gas cluster ion beam secondary ion mass spectrometry
GPXs: Glutathione peroxidases
GPX-4: Glutathione peroxidase-4
GSH: Glutathione
GSSG: Oxidized glutathione
GSSH: Glutathione hydropersulfides
HO-1: Heme oxygenase-1
HSPB-1: Heat shock protein B-1
IREB-2: Iron responsive element-binding protein-2
IRI: Ischemia-reperfusion injury
LOX: Lipoxygenases
LPCAT-3: Lysophosphatidylcholine acyltransferase-3
Lip-1: Liproxstatin-1
LVEF: Left ventricular ejection fraction
MDA: Malondialdehyde
MIRI: Myocardial ischemia-reperfusion injury
MPO: Myoperoxidase
mTOR: Mammalian target of rapamycin
mPTP: Mitochondrial permeability transition pore
NADPH: Nicotinamide adenine dinucleotide phosphate
NOX-4: NADPH oxidase-4
NOX-2: Nicotinamide adenine dinucleotide phosphate oxidase-2
OGD/R: Oxygen–glucose deprivation/reoxygenation
PERK: PKR-like ER kinase
PUFA-PE: Poly-unsaturated fatty acid-phosphatidyl ethanolamine
PEox: Peroxidized phosphatidylethanolamine
ROS: Reactive oxygen species
RSL3: Ras selective lethal compound 3
SIRT-1: Sirtuin-1
STEAP-3: Six transmembrane epithelial antigen of prostate-3
System Xc^−^: Cystine/glutamate antiporter system
TFR-1: Transferrin receptor-1
TLR-4: Toll-like receptor-4
TNF: Tumor necrosis factor
TRAIL: TNF-related apoptosis-inducing ligand
VDAC: Voltage-dependent anion channel.
